# Tumor Microenvironment Uses a Reversible Reprogramming of Mesenchymal Stromal Cells to Mediate Pro-tumorigenic Effects

**DOI:** 10.3389/fcell.2020.545126

**Published:** 2020-11-19

**Authors:** Armel H. Nwabo Kamdje, Paul F. Seke Etet, Richard Simo Tagne, Lorella Vecchio, Kiven Erique Lukong, Mauro Krampera

**Affiliations:** ^1^Department of Physiological Sciences and Biochemistry, Faculty of Medicine and Biomedical Sciences (FMBS), University of Ngaoundéré, Ngaoundéré, Cameroon; ^2^Center for Sustainable Health and Development, Garoua, Cameroon; ^3^Department of Biochemistry, Microbiology and Immunology, College of Medicine, University of Saskatchewan, Saskatoon, SK, Canada; ^4^Section of Hematology, Stem Cell Research Laboratory, Department of Medicine, University of Verona, Verona, Italy

**Keywords:** stromal cells, tumorigenic effects, anticancer effects, tumor microenvironment, reprogramming

## Abstract

The role of mesenchymal stromal cells (MSCs) in the tumor microenvironment is well described. Available data support that MSCs display anticancer activities, and that their reprogramming by cancer cells in the tumor microenvironment induces their switch toward pro-tumorigenic activities. Here we discuss the recent evidence of pro-tumorigenic effects of stromal cells, in particular (i) MSC support to cancer cells through the metabolic reprogramming necessary to maintain their malignant behavior and stemness, and (ii) MSC role in cancer cell immunosenescence and in the establishment and maintenance of immunosuppression in the tumor microenvironment. We also discuss the mechanisms of tumor microenvironment mediated reprogramming of MSCs, including the effects of hypoxia, tumor stiffness, cancer-promoting cells, and tumor extracellular matrix. Finally, we summarize the emerging strategies for reprogramming tumor MSCs to reactivate anticancer functions of these stromal cells.

## Introduction

Mesenchymal stromal cells (MSCs) are multipotent stem cells capable of differentiating into various cell types of the mesodermal lineage, including adipocytes, endothelial cells, fibroblasts, chondrocytes, osteoblasts, and myocytes (Dominici et al., 2006), and possibly into non-mesodermal cell types, such as neural, pancreatic, hepatic, and gastric cells (Oswald et al., 2004; Cislo-Pakuluk and Marycz, 2017; Luo et al., 2020; Xuan et al., 2020). They are a heterogeneous mesenchymal cell population, which resides in the stroma of various tissues and organs and expressing the membrane markers CD105, CD73, and CD90, but not HLA-DR, CD14, CD19, CD31, CD34, and CD45 (Dominici et al., 2006; Nwabo Kamdje et al., 2011). MSCs are a key tool in tissue engineering and regenerative medicine, because they are easily collected and thanks to their ability to migrate and home into damaged tissues where they (i) interact with the microenvironment to drive tissue repair; (ii) transdifferentiate into new cells to restore and/or replace damaged tissues; (iii) rescue organ functions, thanks to their high proliferation, adhesion, migration, differentiation, and immunoregulatory properties (Barberini et al., 2014; Chi et al., 2014; Li et al., 2015). MSC properties are mainly dependent of components of their secretome including numerous factors favoring tissue repair, such as angiopoietin-1 (Ang1), vascular epidermal growth factor (EGF), endothelial growth factor (VEGF), transforming growth factor-beta (TGF-β), hepatocyte growth factor (HGF), fibroblast growth factor (FGF), granulocyte-colony stimulating factor (G-CSF), platelet-derived growth factor (PDGF), interleukin 6 (IL-6) and IL-12, chemokine (C-C motif) ligand 7 (CCL7) and CCL25, and C-X-C motif chemokine 8 (CXCL8), CXCL9, CXCL16, and CCL20 (Ishiki et al., 1992; Li et al., 2013; Wang et al., 2013; Liu et al., 2014; Windmolders et al., 2014; Sesia et al., 2015; Rolandsson Enes et al., 2016; Tsiklauri et al., 2018; Gyukity-Sebestyen et al., 2019; Ozdemir et al., 2019).

However, MSCs also reside in the tumor microenvironment, where they were reported to promote pivotal tumorigenic processes such as: (i) malignant transformation; (ii) cancer cell maintenance and stemness; (iii) cancer stem cell niche formation, including angiogenesis and neovascularization; (iv) metastasis formation; and (v) resistance to anticancer drugs [for review see Seke Etet et al. (2013), Nwabo Kamdje et al. (2014), Atiya et al. (2020), Osman et al. (2020)]. On the other hand, MSC-derived stromal cells restraining cancer growth have been reported in the tumor microenvironment (Bu et al., 2019; Mizutani et al., 2019; Tew et al., 2019) and growing evidence supports that the pro-tumorigenic effects of MSCs emerge from cell reprogramming by the tumor microenvironment (Coffman et al., 2019; Mandal et al., 2019; Al-Jawadi et al., 2020; Boada et al., 2020). Herein, we provide an overview and discuss emerging data supporting MSC reprogramming by the tumor microenvironment and recent reports supporting the existence of stromal cells restraining cancer growth in the tumor microenvironment.

## MSC Pro-Tumorigenic Effects: Immunosuppression and Metabolic Changes

**FIGURE 1:**
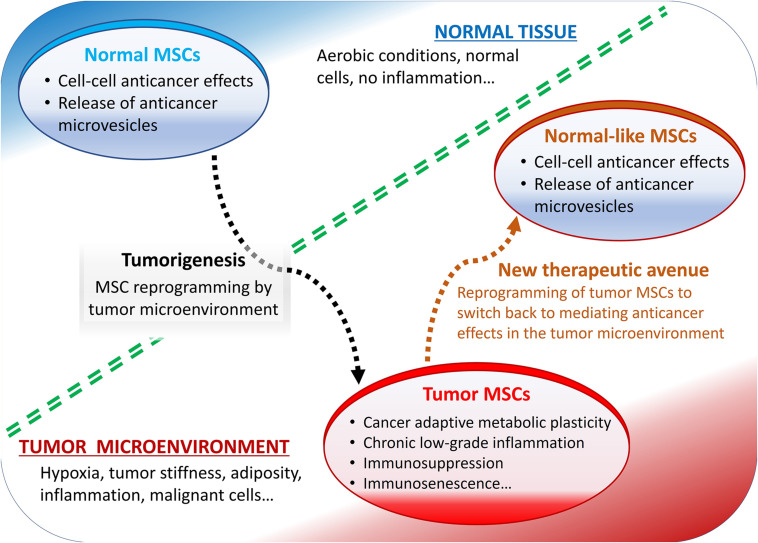
Summary of mesenchymal stromal cells (MSCs) role in the tumor microenvironment. Normally, MSCs mediate anticancer effects but reprogrammed by tumor microenvironment they display pro-tumorigenic effects.

Some of the most reported pro-tumorigenic effects of MSCs include their roles in the metabolic and cellular senescence-like changes, typically observed in various cancers and in the tumor microenvironment-mediated immunosuppression (Figure 1).

### Cancer-Associated Metabolic Changes

The Warburg effect, a metabolic hallmark of tumor cells, is the fact that cancer cells produce most of their ATP via glycolysis, even under aerobic conditions, although it is a less efficient pathway compared to oxidative phosphorylation and despite their greater need for energy [for review see Xu et al. (2015), Fu et al. (2017)]. For instance, pancreatic cancer cells can utilize “metabolic reprogramming,” through the enhancement of glycolysis with increased lactate production and glycolytic enzyme overexpression, to satisfy their energy demand and support malignant behavior, despite a hypoxic and nutrient-deficient microenvironment (Yang et al., 2020). Growing evidence supports that stromal cells trigger the Warburg effect in cancer cells. For instance, the bone marrow (BM)-derived MSCs co-cultured with leukemia cells under normoxic conditions revealed reduced mitochondrial membrane potential and pyruvate metabolism in the both cell types (Samudio et al., 2008). Interestingly, mitochondrial membrane potential reduction was mediated in leukemia cells by an uncoupling protein 2 (UCP2)-dependent mechanism, suggesting that MSCs facilitated the Warburg effect in cancer cells by activating highly conserved mammalian UCPs. On the same hand, a study carried out on bevacizumab-resistant glioblastoma, suggested that inside the hypoxic microenvironment, chemoresistance in cancer cells occurs through: (i) metabolic reprogramming, characterized with suppressed oxidative phosphorylation and upregulated glycolysis; (ii) perivascular invasiveness along remaining blood vessels in a VEGF- and neo-angiogenesis-independent manner; and (iii) enrichment of tumor-initiating stem cells residing in the perivascular niche close to residual blood vessels (Chandra et al., 2020). Such observations are the basis of studies aiming at targeting signaling molecules pivotal for cancer cell glycolysis [for review see Xu et al. (2015)].

An early study aimed at determining whether the Warburg effect is due mainly to the hypoxic microenvironment, or to inherent metabolic alterations in transformed MSCs, revealed that aerobic glycolysis results from MSC oncogenic adaptation to bioenergetic requirements. Authors observed that in some circumstances, transformed MSCs may also rely on increased in oxidative phosphorylation (Funes et al., 2007). However, the study revealed a reversible increase in the transcription of glycolytic enzymes genes, in tumors generated by transformed MSCs, indicating a metabolic support of MSCs for surrounding cells of the tumor to its microenvironment. Similar observations led to the development of a new hypothesis for stromal cell support to cancer cell metabolism, the reverse Warburg effect [for review see Xu et al. (2015), Fu et al. (2017)]. Basically, cancer cells induce oxidative stress in neighboring stromal cells such as MSC-derived fibroblasts by secreting reactive oxygen species (ROS). In stromal cells, ROS trigger aerobic glycolysis and the production of lactate, pyruvate, and other high energy metabolites. Finally, the latter are transported to adjacent cancer cells where they sustain the energy need and various signaling pathways driving tumor progression, metastasis, and chemoresistance (Fu et al., 2017). This new approach focused on tumor cell metabolism, pointed out many targets in tumor microenvironment and cancer cell machinery for anticancer therapy.

A wealth of other reports also supports the involvement of stromal cells in the metabolic changes supporting tumorigenic processes. For instance, In breast cancer, depending on ROS, hypoxia, and glucose availability in the microenvironment, tumor-initiating cells are capable to switch toward oxidative phosphorylation and glycolysis. This adaptative metabolic switch is controlled at least in part by stromal cells to confer a survival advantage to malignant cells (Walsh et al., 2019). Lung et al. (2019) reported that the expression of estrogen receptor-α (ER), the target of endocrine therapies in breast cancer, is regulated by the BM microenvironment. In this study, the induction of ESR1 mRNA and ER protein downregulation, through a MAPK-independent mechanism, was achieved by the treatment of breast cancer cells with conditioned culture media from either cancer-activated BM stromal cells or HS5 BM stromal cell line (Lung et al., 2019). In addition, thyroid hormones, which are well-established pro-tumorigenic players, may stimulate tumor growth and neovascularization in various solid cancers by activating MSCs through a non-classical integrin αvβ3 signaling (Schmohl et al., 2019). Moreover, the EGF-like superfamily member EGFL6 would promote tumor growth by mediating a cross-talk between stromal and cancer cells that would contribute to stemness and epithelial-mesenchymal transition (EMT), an important tumorigenic mechanism where epithelial cells become MSCs by losing their cell polarity and adhesion ability, and by gaining migratory and invasive properties (An et al., 2019). Altogether, these observations confirm that targeting cancer cell energy metabolism is still a sound anticancer strategy, and point out stromal cells as major players in cancer cell energy metabolism.

### Immunosenescence and Immunosuppression

Immunosuppression and immunosenescence are two major immunological phenomena observed in the tumor microenvironment. Like aging processes, cancers environment is characterized by a chronic inflammation (“inflammaging”) and cellular senescence (“immunosenescence”). The role of stromal cells’ immunomodulation in shaping a senescent microenvironment in broad spectrum of human malignancies, especially tumorigenesis, has been documented extensively [for review see Salminen et al. (2020), Thomas et al. (2020)]. For instance, BM stromal cells from patients with myelodysplastic syndrome display a senescence phenotype induced by S100A9-induced Toll-like receptor 4 (TLR4), NLRP3 inflammasome activation and IL-1β secretion (Shi et al., 2019). Senescent breast luminal cells promoted carcinogenesis by activating MSC-derived fibroblasts through the inflammatory cytokine IL-8 (Al-Khalaf et al., 2019). Acute myeloid leukemia (AML) blasts induced a senescence-associated secretory phenotype (SASP) in BM stromal cells through a p16INK4a-dependent mechanism, which encompassed the irreversible arrest of cell proliferation and the secretion of a set of chemokines, proinflammatory cytokines, and growth factors (Abdul-Aziz et al., 2019). MSCs promoted the progression of gastric cancer cells through the release of CXCL16, which activates STAT3-mediated expression of Ror1 in the cancer cells (Ikeda et al., 2020). In oral mucosa carcinogenesis, MSCs increased immunosuppressive functions on T cell proliferation and pro-tumorigenic effects of tumor resident MSCs correlated with higher expressions of cellular proliferative status indicator Ki67 (Chen et al., 2019a). STAT4 over-expression in gastric cancer cells made normal fibroblasts acquire cancer-associated fibroblast (CAF)-like features through wnt/β-catenin-dependent signaling (Zhou et al., 2019a). A recent study using MSCs expanded from BM and prostate cancer tissue from independent donors showed that tumor-infiltrating MSCs are major drivers of the immunosuppressive tumor microenvironment in prostate cancer (Krueger et al., 2019). The authors reported the ability of prostate cancer-infiltrating MSCs to suppress T-cell proliferation through immunosuppressive properties comparable to canonical BM-derived MSCs. The suppression of proliferation mediated by prostate cancer-infiltrating MSCs was dose-dependent, and the expressions of PD-L1 and PD-L2 were upregulated on T-cells in the presence of IFN-γ and TNF-α (Krueger et al., 2019). In another study, the transcriptome analysis of MSCs from multiple myeloma patients revealed constitutive abnormalities in immune system activation, cell cycle progression, and osteoblastogenesis that were maintained even in the absence of tumor cells, thus strongly suggesting that MSCs may contribute to the immune evasion and bone lesions frequently found in multiple myeloma (Fernando et al., 2019). Altogether, these observations also point out stromal cells as major players in tumorigenesis and reveal more targets for pharmacological anticancer therapy.

Although it is well-established that MSCs are major drivers of the immunomodulation observed in solid tumor microenvironment, many others cell components can cooperate with MSCs to modulate immune response. In AML, CXCL8 supports the survival and proliferation of leukemic cells via the PI3K/AKT signaling pathway. In the affected BM microenvironment CXCL8 is mainly secreted by MSCs (Cheng et al., 2019). Study on other bone marrow disorders show that and MSCs to shape the Microenvironment at least partly by inducing suppressive monocytes and dampening NK cell functions (Sarhan et al., 2020).

## Evidence of Stromal Cell Programming by Tumor Microenvironment

A growing body of evidence supports that stromal cells follow the program dictated by their microenvironment.

### Tumor Microenvironment Effects on Stromal Cells

Early studies addressing the composition of the tumor microenvironment, reported an atypical cellular and molecular microenvironment supporting carcinogenesis and chemoresistance (Garcia-Hernandez et al., 2017; Pelizzo et al., 2018). Recently, Coffman et al. (2019) reported that ovarian carcinoma-associated MSCs, which are critical stromal progenitor cells promoting tumor cell growth, cancer stemness and chemoresistance, arose from a process of tumor-mediated reprogramming of local tissue MSCs. This study also provided strong evidence that tumor-mediated MSC conversion is tissue- and cancer-type dependent, requiring tumor-secreted factors and hypoxia (Coffman et al., 2019). In other studies, gene expression signatures and mesenchymal shift in quiescent glioblastoma cells, a source of tumor recurrence in highly malignant glioblastoma, was observed following their interactions with niche microenvironment (Tejero et al., 2019). Breast tumor microenvironment transformed naive MSCs into tumor-forming cells in nude mice. Indeed, MSCs pre-exposed to conditioned medium or purified exosomes derived from breast cancer cells (MDA-MB-231) formed a tumor-like mass rich in stromal tissue by 14 weeks when injected into mammary glands of nude mice (Worner et al., 2019). Similarly, CCL5 secreted by classic Hodgkin lymphoma cells recruited MSCs and monocytes, enhancing MSC proliferation and CCL5 secretion. Conditioned medium from these educated MSCs increased tumor cell growth and monocyte migration (Casagrande et al., 2019). Similarly TLR4 signaling educated MSCs to promote tumor microenvironment transformation in multiple myeloma (Giallongo et al., 2019).

Exosomes including extracellular vesicles (EVs) represent a mean used by tumor cells to educate MSCs in the microenvironment. In chronic myeloid leukemia (CML), leukemia cells altered the cellular and immune-related properties of BM-MSCs and macrophages *in vitro* by the mean of exosomes (Jafarzadeh et al., 2019). Consistently, Zannoni et al. (2019) reported that EVs released by monocytes from chronic myelomonocytic leukemia patients conferred a procoagulant state favorable for cancer progression, through a tissue factor-dependent mechanism mediated by MSCs. In glioma, exosomes from cancer cells induced a tumor-like phenotype in MSCs by activating glycolysis (Ma et al., 2019). In gastric cancer, tumor cell-derived exosomes affected the immunomodulatory functions of MSCs by activating the NF-kB signaling pathway, which in turn mediates support to tumor growth by maintaining the inflammatory environment and enhancing the ability of MSCs to activate immune cells (Shen et al., 2019).

In addition, pre-metastatic niche in distant organs may be created, at least in part, by the transfer of EVs secreted by tumor-associated macrophages (TAMs) to stromal cells, such as fibroblasts, peritoneal mesothelial cells (PMCs), and endothelial cells (Umakoshi et al., 2019). Long-term culture of human MDA-MB-231 breast cancer cells with normal human MSCs was associated with the formation of three-dimensional (3D) tumor spheroids *in vitro*, with a 14-fold enhanced expression of the breast tumor marker urokinase plasminogen activator (uPA; Melzer et al., 2019). Similarly, MSCs cultured with colorectal cancer cells showed increased invasiveness and proliferative abilities due to increased TGF-β1 and decreased p53 levels (Oh et al., 2020). In another study, TGF-β1 promoted the migration and invasion of HCT116 and HT29 colorectal cancer cells, and induced the differentiation of MSCs into CAFs through a JAK/STAT3 signaling-depended mechanism (Tan et al., 2019).

The available data also support detrimental cross-talks between stromal and cancer cells. For instance, reciprocal reprogramming of cancer stem cells (CSCs) and associated MSCs may promote tumor progression in gastric cancer (Shamai et al., 2019). Similarly, asporin, a factor secreted by MSCs following cellular interactions within the tumor microenvironment, altered the tumor microenvironment and inhibited MSC differentiation to drive metastatic progression through CD49d/CD29 signaling (Hughes et al., 2019). Moreover, Dabbah et al. (2019) reported that microvesicles derived from BM MSCs of multiple myeloma patients increased the tumorigenicity of MM cells (Hughes et al., 2019). In this study, CD49d and CD29 integrin overexpression in MM-MSC microvesicles were associated with patient staging and response to treatment. The concomitant inhibition of these molecules resulted in reduced uptake of EVs by MM-MSC (but not normal donor MSC microvesicles), and downregulation of aggressiveness markers, thus enhancing response to chemotherapy (Dabbah et al., 2019). Interestingly, this study also suggested that the reciprocal interactions of malignant cells and MSCs in breast cancer microenvironment may result in the transformation of naive MSCs into cells capable of forming explants in nude mice (Dabbah et al., 2019).

Overall, together with evidence of MSC role in cancer metabolism discussed in section 1.1, these data suggest that tumorigenesis is triggered and driven by a bidirectional cross-talks between MSCs and tumor environment. Therefore, unraveling the signaling molecules involved in these pro-tumorigenic cross-talks may lead to the identification of novel targets for anti-cancer therapy. Promising reports using this approach include a recent study that addressed the potential roles and mechanisms of long non-coding RNAs in EMT and in the maintenance of CSC-like properties in non-small cell lung cancer (NSCLC). Using A549 and H1299 human NSCLC cell lines, L9981 and 95D highly metastatic NSCLC cell lines, and NL9980 and 95C low-metastatic NSCLC cell lines., the authors observed that knockdown of long non-coding RNA linc-ITGB1 inhibited the expression of various markers of cancer stemness and CSC formation by reducing the expression of the EMT-related transcription factor Snail. Rewardingly, the overexpression of Snail reversed the inhibitory effects of linc-ITGB1 knockdown (Guo et al., 2019). Further studies should characterize and target the signaling pathways supporting the reprogramming of stromal cells by cancer cells, as well as other interactions between these cells that support tumorigenesis.

### Hypoxia and Tumor Stiffness

Tumor stiffness and hypoxia are key conditions of the solid tumor microenvironment, known to promote tumor survival, progression and metastasis. Hypoxia-driven phosphorylated glycoprotein such as osteopontin, promoted stem cell-like properties and EMT in pancreatic cancer cells in a paracrine manner, through integrin αvβ3-Akt/Erk- forkhead box protein M1 (FOXM1) signaling (Cao et al., 2019). Hypoxia-induced EMT was observed in non-small-cell lung cancer (Chen et al., 2019b) where hypoxia induced the acquisition of cancer stem cell features through CXCR4 activation (Kang et al., 2019). In fact earlier reports pointed out HIF-1 as a link between hypoxia, inflammation, and cancer [for review see Balamurugan (2016), Shi et al. (2018)]. Growing evidence suggests that stromal cells mediate the pro-tumorigenic effects of hypoxia and tumor stiffness. Notably, MSC-derived CAFs were suggested as the link between biophysical forces and pro-metastatic signaling in colon cancer, as they respond to increased stiffness of the tumor microenvironment by activating TGF-β family members and the signaling of the strong pro-metastatic cytokine activin A (Bauer et al., 2020). On the same hand, microvesicles derived from human BM-MSCs supported human osteosarcoma (U2OS) cell growth under hypoxia conditions both *in vitro* and *in vivo* through PI3K/AKT and HIF-1α-dependent mechanisms (Lin et al., 2019). In addition, interactions of cancer cells and stromal cells in hypoxic microenvironment were found to drive EMT through NOTCH and c-MET signaling, inducing an immunosuppressive response within the microenvironment in pancreatic ductal adenocarcinoma (PDA; Daniel et al., 2019).

In a study addressing the end-stage of myeloma cell mobilization from the BM into peripheral blood (PB), hypoxic BM niches, together with a pro-inflammatory microenvironment resulting from the interactions between tumor cells and BM stromal cells, were able to induce an arrest in proliferation, thus forcing tumor cells to circulate into the peripheral blood to seek other BM niches (Garces et al., 2020). These observations suggest that hypoxic BM niches are key players in metastatic processes. In agreement with this view, it has been observed in an *in vivo* mouse syngeneic tumor model, that hypoxic BM stromal cells-derived exosomal miRNAs promoted metastasis of lung cancer cells via STAT3-induced EMT (Zhang et al., 2019a,b). Consistently, Saforo et al. (2019) described an *in vitro* cell culturing system incorporating elements of the *in vivo* lung environment, including physiological hypoxia (5% O2) and lung fibroblast-derived extracellular matrix. Through this culture system, a rapid expansion of stromal progenitors from patient’s lung tumor resections was achieved. These progenitor cells retained the secretion of factors associated with cancer progression, the expression of pluripotency markers, and the ability to enhance tumor cell growth and metastasis (Saforo et al., 2019). The ability of hypoxia-conditioned MSCs to promote cancer progression was also observed in hepatocellular carcinoma but the effect was dependent of yes-associated protein (YAP)-mediated lipogenesis reprogramming (Liu et al., 2019). In Glioblastoma, the glioblastoma stem-like cells (GSCs) phenotype, the worst prognostic marker of Glioblastoma, was reported to persist due to hypoxic microenvironment-dependent release of extracellular adenosine, which in turn, promote cell migration, invasion and tumor recurrence through the activation of the A3 Adenosine Receptor (A3AR; Torres et al., 2019).

Altogether, these data suggest that hypoxia, tumor stiffness, and inflammation are among the major drivers of the pro-tumorigenic reprogramming of stromal cells in the tumor microenvironment.

### Extracellular Matrix Involvement

Emerging data strongly suggest that the tumor extracellular matrix (ECM) also contributes to tumor microenvironment effects on stromal cells. For example, after showing that multiple myeloma (MM) cells, cultured with BM-MSCs, co-modulated the phenotype of MM cells in a MAPKs/translation initiation (TI)-dependent manner, Ibraheem et al. (2019) reported that even the decellularized ECM of BM-MSCs from MM patients was able to induce comparable pro-tumorigenic effects (Ref). A number of changes in microRNAs was shown to affect MM phenotype and the activation of MAPK/TI, EMT, proliferation, and CXCR4, with a role for BM-MSCs secretome and microvesicles. On the other hand, the decellularized ECM of BM-MSCs from normal donors mediated anticancer effects, including a rapid and persistent decrease in MAPK/TI activation, proliferation, cell count, viability, migration, and invasion (Ibraheem et al., 2019). These authors also provided evidence of a synergism between the ECM and microvesicles in the modulation of MM cell response to chemotherapy as well as in the hierarchy and interdependence of MAPKs/TI/autophagy/phenotype cascade. These observations suggested that to reprogram MSCs for pro-tumorigenic effects, the ECM also needs to be reprogrammed by cancer-promoting cells. For example, senescent MSCs actively remodeled the surrounding ECM to drive breast cancer cells to a more-invasive phenotype (Ghosh et al., 2020). Consistently, matrix metalloproteinase-9 (MMP-9) produced by leukemia cells facilitated tumor progression via remodeling of the ECM of the BM microenvironment. This is supported by the fact that MMP-9-deficiency in the BM microenvironment reduced leukemia-initiating cells and prolonged survival of mice with BCR-ABL1-positive B-cell acute lymphoblastic leukemia (B-ALL; Verma et al., 2020).

Three-dimensional culture studies with cancer and stromal cells in ECM and multiplex quantitative analysis method, reprensent majors tool to tacle signaling molecules and mechanisms used by reprogrammed ECM to drive MSC pro-tumorigenic effects, (Hwang et al., 2019; Maliszewska-Olejniczak et al., 2019). Therefore, a recent study using such approach in hepatocellular carcinoma (HCC), showed that cell repopulation of cirrhotic scaffolds displayed a unique up-regulation of genes related to EMT and TGF-β signaling as well as high concentration of endogenous TGF-β1 in comparison to healthy scaffolds and TGF-β1-induced phosphorylation of canonical proteins Smad2/3 (Mazza et al., 2019). This study characterized the inherent features of ECM microenvironment from human cirrhotic liver acting as key pro-tumorigenic components in HCC development.

### Impact of Adiposity

It is well-established that fat tissue overgrowth in obesity promotes tumor progression [for reviews of earlier reports see Park et al. (2014), Iyengar et al. (2016), Quail and Dannenberg (2019)]. Using a xenograft model of early multiple myeloma, it has been shown that bone niche switching towards a “fatty” marrow supports the development of malignant cells during carcinogenesis. In this study, MSCs mainly gave rise to adipocytes supporting tumor growth by increasing the survival and chemoresistance of malignant cells (Berlier et al., 2019). Su et al. (2019) compared lean and obese mice grafted with prostate tumors and showed that obesity promotes EMT in cancer cells and tumor invasion into the surrounding fat tissue. In this study, adipose stromal cells induced EMT in prostate cancer cells and rendered them more migratory and chemo-resistant. By contrast, interference of adipose stromal cell capabilities suppressed both EMT and chemoresistance to docetaxel, cabazitaxel, and cisplatin chemotherapy in human prostate cancer cells (Su et al., 2019). It has been suggested that that adipose-derived factors may play a role in MSC-mediated pro-tumorigenic effects. For instance, the adipokine chemerin, a cell differentiation promoter and leukocyte chemoattractant factor established as a major player in obesity-mediated support of cancer progression, was reported to promote the growth, proliferation migration, invasion, and metastasis of cancer cells. The effects of the adipokine chemerin were achieved through the recruitment of tumor-associated MSCs and the stimulation of angiogenesis pathways in endothelial cells through chemerin receptor 1 (CMKLR1), chemerin receptor 2 (GPR1), and CCLR2 signaling (Goralski et al., 2019). Interestingly, in a culture system established to investigate the paracrine effects of MSCs on the migration and invasion potential of this aggressive breast cancer cell line, human adipose-derived MSCs promoted EMT in MCF7 breast cancer cells by cross-interacting with the TGF-β/Smad and PI3K/AKT signaling pathways, suggesting that stromal cells are key players in obesity-mediated tumor progression (Wu et al., 2019). There is probably a pro-tumorigenic cross-talk between adipose tissue and tumor stromal cells, particularly in obesity-like contexts. Adipose-derived signaling molecules might be among the drivers of pro-tumorigenic reprogramming of stromal cells by cancer cells. The evidence of stromal cell programming by tumor microenvironment suggest that non programmed cells could mediate anticancer effects in contrast with their educated counterpart.

## Evidence of Stromal Cells Mediating Anticancer Effects

Emerging data support that non-tumor associated MSCs mediate anticancer effects and suggest the existence of stromal cells mediating anticancer effects in the tumor microenvironment, notably, MSCs slowing tumor progression and cancer-restraining CAFs.

### Naïve Stromal Cells Mediate Anticancer Effects

To address the antitumor potential of non-tumor associated-MSCs, Francois et al. (2019), treated immunocompetent rat models of colorectal carcinogenesis with non-tumor BM-derived MSCs, observing inhibition of cancer progression. This effect was partially due to the control of the tumor microenvironmental immunity as shown by (i) the modulation of effector cells, such as regulatory T cells (Tregs), CD8+ cells and NK cells; (ii)macrophage reprogramming into regulatory cells performing phagocytosis with reduced production of proinflammatory cytokines; (iii) the restoration ofTh17 activity and (iv) 50% decrease in the infiltration rate of CD68+ cells, and two-fold increase of CD3+ cells (Francois et al., 2019). In another study, intra-BM but not the systemic administration of BM MSCs from healthy donors reduced tumor burden and prolonged survival of the leukemia-bearing mice (Xia et al., 2020). In this study, the MSC senescence observed during disease progression was stopped and the BM microenvironment was restored, with functional recovery of host myelopoiesis and improvement of thrombopoiesis. Moreover, in a bioluminescence imaging study monitoring the effects of human umbilical cord-derived MSCs in mouse hepatoma tumor models with H7402 cell line, MSC microenvironment effectively inhibited the growth of cancer cells (Liu et al., 2019). Interestingly, human BM MSC-derived exosomes overexpressing miR-34a inhibited glioblastoma development (Wang et al., 2019). Two other microRNAs, associated with the capacity of MSCs to attenuate cancer growth have also been identified, namely miR-150 and miR-7 (Wang et al., 2019). Mandal et al. (2019) reported that perinatal tissue MSCs encapsulated with the sodium alginate biomaterial for isolation from tumor microenvironment displayed: (i) increased proliferation with enhanced expressions of pluripotency genes, EMT, immune-modulation and angiogenesis; (ii) increased expression of the tumor invasion suppressor protein E-cadherin; (iii) and increased secretions of VEGF, TGF-β, TNF-α, IFN-γ, IL-10 and IL-6, and IL-3β. Furthermore, treatment of CSCs derived from MDA-MB-231 and MCF7 breast cancer cell lines with encapsulated MSCs lowered CSC viability and migration, with downregulation of markers related to angiogenesis, EMT and proliferation, and upregulation of Wnt antagonists sFRP4 and DKK1 (Mandal et al., 2019). Taken together, these data suggest that non-tumor associated MSCs mediate anticancer effects and support that MSC pro-tumorigenic effects result from tumor reprogramming.

Early clinical and experimental studies in mouse models suggested the existence of at least two types of MSC-derived CAFs: they extensively studied cancer-promoting CAFs and the cancer-restraining CAFs, which were poorly investigated due to the lack of markers [for review see Bu et al. (2019)]. In a recent study using stromal cell lines derived from central nervous system (CNS) metastasis of breast and lung cancer patients, a cell population with tumor inhibitory functions, expressing high levels of collagen and displaying gene expression signatures of CAFs, MSCs, and EMT, was isolated and characterized in cancer metastasis microenvironment (Tew et al., 2019). Some very interesting recent reports have proposed markers to identify cancer-restraining CAFs. The study of Mizutani et al. (2019) has reported the glycosylphosphatidylinositol-anchored protein Meflin as a potential marker of cancer-restraining CAFs. These authors observed that the tissue infiltration of Meflin-positive CAFs correlated with favorable patient outcome in pancreatic ductal adenocarcinoma. Meflin deficiency or downregulated resulted in markedly faster tumor progression in a pancreatic ductal adenocarcinoma mouse model. Consistently, the overexpression of Meflin in CAFs or the delivery of a Meflin-expressing lentivirus into the tumor stroma were sufficient to suppress the growth of xenograft tumors (Mizutani et al., 2019). This new marker paves the way to isolation and further characterization of CAFs exerting anti-tumoral effects.

Overall, *in vitro* studies and studies using naïve MSCs, i.e., MSCs that were not in contact with tumor microenvironment, support the anti-tumor effects of MSCs. But these anti-cancer functions can be markedly reduced by the direct crosstalk with tumor bulk or tumor stromal elements. Interestingly, Early studies addressing the immunological hallmarks of MSCs in the tumor microenvironment revealed various molecular mechanisms through which MSCs may modulate the immune response in the cancer microenvironment. This indicated that it may be possible to convert the microenvironment from immunosuppressive to immunostimulant feature [for review see Turley et al. (2015), Poggi et al. (2018)]. These reports paved the way for studies attempting to reprogram tumor stromal cells for anticancer effects.

### Attempts to Reprogram Tumor Stromal Cells for Anticancer Effects

A bulk of recent reports propose promising strategies for reprogramming microenvironmental stromal cells to mediate only anticancer effects. For instance, treatments with various flavonoids and non-flavonoid polyphenolic compounds from medicinal plants alleviated multidrug resistance in breast, prostate, lung and colorectal cancer with survival benefits in patients. These effects were achieve through the modulation of inflammatory responses, their antioxidant capacity, and the inactivation of oncogenes, the inhibition of angiogenesis, proliferation, survival, and metastasis [for review see Costea et al. (2020)]. On the same hand, unlike conditioned medium from human adipose MSCs, eicosapentanoic acid-treated adipose MSCs reduced mRNA levels of the tumor-associated genes FASN, STAT3, cIAP-2 in MDA-MB-231 and MCF-7 breast cancer cell lines. Functionaly, cancer cell lines treated in these conditions displayed reduced glycolysis, inflammation and motility *in vivo* (Al-Jawadi et al., 2020). In addition, treatment with 5-Azacytidine restored IL-6-increased production in MSCs collected from myelodysplastic patients (Boada et al., 2020). Engineered human placenta-derived MSCs, armed with a double fusion gene containing the herpes simplex virus truncated thymidine kinase and firefly luciferase, inhibited the tumorigenesis mediated by the HT29 colon cancer cell line in nude mice (Yang et al., 2019). Similarly, as compared to short-culture CAFs, prolonged culture of heterogeneous prostatic CAFs resulted in marked decreases in the expression of proliferative endothelial cell surface marker endoglin (CD105), and loss of their tumor expansion potential in 3D-cultures and patient-derived xenograft tissues (Kato et al., 2019).

Furthermore, irradiated endothelial cells decreased the malignancy of liver cancer cells in a culture system using conditioned medium from endothelial cells, suggesting that irradiated endothelial cells are key players in the therapeutic effects of radiotherapy (Kim et al., 2019). Similarly, a study addressing the response of human MSCs to low-dose photodynamic therapy revealed that this treatment may increase MSC immunogenicity and promote angiogenic potential (Udartseva et al., 2019). In this *in vitro* study, low-dose photodynamic therapy: (i) induced the reorganization of MSC cytoskeleton, with decrease in cell motility; (ii) induced the inhibition of GSK-3 and the activation of Erk1/2 signaling in MSCs; (iii) significantly upregulated the secretion of VEGF-A, IL-8, PAI-1, MMP-9, and other proangiogenic factors by MSCs; (iv) dramatically inhibited the secretion of pro-tumorigenic macrophage infiltration marker CCL2 (MCP-1) by MSCs and decreased MSC viability and immunogenicity when cultured with lymphocytes. In another study, MSCs loaded with photosensitizer MnO2@Ce6 successfully shipped these nanoparticles into lung cancer tumor sites, enhancing the effects of photodynamic therapy *in vivo* (Cao et al., 2020). In sarcomas, tumor-initiating cells are thought to derive from MSCs, modified MSC were successfully used to deliver TNF-related apoptosis-inducing ligand (TRAIL) to induce tumor apoptosis, open novel therapeutic opportunities (Grisendi et al., 2015). Altogether, these observations confirm that it is possible to reprogram tumor stromal cells to mediate anticancer effects, and warrant further studies aimed at developing therapies using this approach. We propose that reprogrammed tumor MSCs loaded with photosensitizer MnO2@Ce6 or other nanoparticles with anticancer effects could display strong anticancer effects *in vivo*, as this approach will couple the MSC anticancer effects with the anticancer effects of nanoparticles (Ref).

## Controversy Sources and Implications for MSC Therapeutic Use

### Other Sources of Controversy on the Roles of Stromal Cells in Tumorigenesis

Beyond the fact that MSC effects in the tumor microenvironment depend on the interactions with the adipose tissue and malignant cells (pro-tumorigenic when there are cross-talks and anticancer effects when there are poor interactions) and with the ECM programming (pro- or anticancer) to support differences between *in vitro* and *in vivo* studies, controversies on the roles of stromal cells in the tumor microenvironment may also emerge from MSC origin and the cancer type. This hypothesis is fully supported by a report of Quach et al. (2019) where the inhibition of the glypican-1 (GPC-1) prostate cancer biomarker in the aggressive prostate cancer cell line PC-3 decreased cell growth and migration *in vitro*, but increased PC-3 tumor size in NCr nude mice xenografts. Authors also observed that GPC-1 inhibition in an aggressive prostate cancer cell line, the DU-145 cells, increased cancer cell proliferation and migration, suggesting that GPC-1 accounts among the factors that drive cancer cell line-dependent responses to stromal cells Reduced cell growth observed in GPC-1 knockdown PC-3 cells was rescued by culturing the cells with MSCs and CAFs. Further, the treatment of these stromal cells with tumor-conditioned media from PC-3 cells transfected with GPC-1 shRNA increased the expression of extracellular matrix components, endocrine and paracrine biomolecules, and migration markers (Quach et al., 2019). In another study, despite *in vivo* observations revealing the ability of IGF/IGF-IR signaling to induce drug resistance and influence the ability to form metastasis via the induction of EMT in pancreatic cancer, the activation of this signaling pathway by stromal cells failed to induce EMT in cultures with MiaPaCa-2, AsPC-1, Capan-2, BxPC-3, and Panc1 pancreatic cancer cell lines (Kopantzev et al., 2019), suggesting a key role for tumor microenvironment for the pro-tumorigenic effects of this MSC-activated signaling pathway.

When assessing how breast cancer cells from different stages of the metastatic cascade convert MSCs into tumor-associated MSCs, it was observed that only MDA-MB-231 breast cancer secretomes, but not MCF-7 cells or sublines isolated from bone, lung, and brain metastases, converted MSCs into tumor-associated MSCs in bioengineered 3D microenvironments (Blache et al., 2019). These observations further confirm that MSCs from tumor microenvironment are reprogrammed by cancer-initiating cells and primary tumor ECM to mediate pro-tumorigenic effects, and that without such reprogramming the stromal cells may rather mediate anticancer effects. On the same hand, in co-cultures with stem cell-like (CD133+) cells from urinary bladder cancer cell lines, adipose-derived MSCs produced soluble mediators that: (i) increased the phosphorylation of molecules involved in cancer progression and drug resistance, such as p70 S6K, ERK1/2, and AKT1/2/3 in CD133+ cells (5,637 cell line); but instead, (ii) decreased the phosphorylation of those involved in PI3K/Akt and MAPK signaling molecules in CD133+ cells (HB-CLS-1 cell line; Maj et al., 2019). In this study, there seemed to be a controversy on the effect of MSCs on urinary bladder cancer lines *in vitro*, as MSCs induced pro-tumorigenic effects in culture with 5637 cell line and anticancer effects in culture with HB-CLS-1 cell line. However, this difference may actually suggest that unlike the first, the latter cell line was not able to reprogram MSCs for pro-tumorigenic effects, hence, MSCs mediated anticancer effects.

However, considering that naïve MSCs promoted anticancer effects in most reports, treatment of MDAMB231 and MCF7 human breast cancer cells with medium containing EVs from naïve MSC cultures promoted the *in vitro* proliferation and migration of cancer cells through ERK signaling (Zhou et al., 2019b). We hypothesize that these effects may be due to differences in the origin of MSCs, as unlike in most reports, in this study human umbilical cord MSCs, and not BM or adipose-derived MSCs were used. A comparative study of subcutaneous and visceral adipose-derived MSCs revealed various functional similarities and differences, despite similar surface markers (Ritter et al., 2019). Notably, visceral MSCs secreted higher levels of inflammatory cytokines (IL-6, IL-8, and TNF-α) and had more active sonic hedgehog pathway than subcutaneous MSCs. Moreover, fetal and adult lung MSCs possess lung-specific properties, unlike BM MSC (Rolandsson Enes et al., 2016). A study profiling the transcriptomes of 361 single MSCs derived from two umbilical cords (UC-MSCs), harvested at different passages and stimulated with or without inflammatory cytokines, revealed that UC-MSCs are a well-organized population with limited heterogeneity, as compared to other MSC types (Huang et al., 2019). These data support strong differences between MSC lines based on their origin, and even raise caution for the therapeutic use of some MSC lines in cancer context.

### Implications for MSC Use for Tissue Regeneration in Cancer Patients

Because MSCs are able to increase cancer cell malignancy *in vitro*, early studies raised the danger of the application of human MSCs in regenerative medicine for patients with history of breast cancer, small cell ovarian cancer and other malignancies (Kucerova et al., 2011; Yang et al., 2015). Although subsequent reports from *in vitro* studies provided encouraging results for potential use of MSCs from patients for post-anticancer therapy tissue regeneration, there are still some concerns. For instance, an *in vitro* biosafety profile evaluation of MSCs derived from the BM of sarcoma patients showed that the *in vitro* expansion of MSCs from osteosarcoma (OS) and Ewing sarcoma (EWS) patients does not favor malignant transformation, but instead of that these MSCs displayed comparable morphology, immunophenotype, differentiation potential, proliferation rate, and telomerase activity to MSCs from healthy donors, indicating that OS and EWS patients may benefit from an autologous MSCs-based bone reconstruction after anticancer chemotherapy (Lucarelli et al., 2014). However, these promising findings, which need to be confirmed *in vivo*, were mitigated by the observation of chromosomal aberrations in MSCs after culture, raising caution and confirming the need for rigorous phenotypic, genetic and functional evaluation of the biosafety of MSCs from patients before clinical use. Interestingly, reports from exploratory studies in mice confirmed the therapeutic potential of MSCs for repairing damaged tissues after anticancer chemotherapy *in vivo*, thus after elimination of most of the primary tumor tissue that could have reprogrammed MSCs to mediate pro-tumorigenic effects [for review see Bussard et al. (2016), Baghban et al. (2020)]. Notably, human adipose-derived MSCs displayed repairing properties in damaged thymus following chemotherapy in mouse models of blood cancer, with improvements in the thymic structure and functions, as shown by the proportion of circulating and splenic Treg cells and the recovery of T-cell subpopulations (Zhan et al., 2019). However, the caution remains, not because of the possibility of reprogramming of MSCs for pro-tumorigenic effects, considering that anticancer chemotherapy normally eliminates most of the primary tumor tissue, but because we still need studies proving phenotypic, genetic and functional biosafety of MSCs in cancer context. Autologous MSC use may require a biosafety evaluation for each patient considering the clinical implications of using damaged MSCs.

## Concluding Remarks

The available data support that stromal cells normally have anticancer cancer effects. MSCs reprogrammed by cancer cells in the tumor microenvironment undergo a switch towards pro-tumorigenic activities, including their support to cancer cells in part through the metabolic reprogramming necessary to satisfy the energy demand and malignant behavior of the latter in a hypoxic and nutrient-deficient microenvironment. Tumor microenvironment reprograms MSCs thanks to hypoxia and the extracellular matrix cross-talks with MSCs. Interestingly, promising emerging reports suggest strategies for reprogramming microenvironmental stromal cells, which in turn switch back to naïve MSCs capable to function as anti-cancer agents. These reprogramming treatment include MSCs treatment with polyphenolic compounds from medicinal plants, with eicosapentanoic acid, or with 5-Azacytidine…. Taken together, the available data suggests that targeting the tumor microenvironment could be a promising therapeutic strategy in cancer, and that it is possible to reprogram tumor stromal cells to revert back to anticancer effects. These strategies should be further developed in the search for anticancer therapies, in particular for refractory cancers.

## Author Contributions

All authors listed have made a substantial, direct and intellectual contribution to the work, and approved it for publication.

## Conflict of Interest

The authors declare that the research was conducted in the absence of any commercial or financial relationships that could be construed as a potential conflict of interest.
